# SUP: an extension to SLINK to allow a larger number of marker loci to be simulated in pedigrees conditional on trait values

**DOI:** 10.1186/1471-2156-7-40

**Published:** 2006-07-03

**Authors:** Mathieu Lemire

**Affiliations:** 1 McGill University and Genome Quebec Innovation Centre, Montreal, Canada

## Abstract

**Background:**

With the recent advances in high-throughput genotyping technologies that allow for large-scale association mapping of human complex traits, promising statistical designs and methods have been emerging. Efficient simulation software are key elements for the evaluation of the properties of new statistical tests. SLINK is a flexible simulation tool that has been widely used to generate the segregation and recombination processes of markers linked to, and possibly associated with, a trait locus, conditional on trait values in arbitrary pedigrees. In practice, its most serious limitation is the small number of loci that can be simulated, since the complexity of the algorithm scales exponentially with this number.

**Results:**

I describe the implementation of a two-step algorithm to be used in conjunction with SLINK to enable the simulation of a large number of marker loci linked to a trait locus and conditional on trait values in families, with the possibility for the loci to be in linkage disequilibrium. SLINK is used in the first step to simulate genotypes at the trait locus conditional on the observed trait values, and also to generate an indicator of the descent path of the simulated alleles. In the second step, marker alleles or haplotypes are generated in the founders, conditional on the trait locus genotypes simulated in the first step. Then the recombination process between the marker loci takes place conditionally on the descent path and on the trait locus genotypes. This two-step implementation is often computationally faster than other software that are designed to generate marker data linked to, and possibly associated with, a trait locus.

**Conclusion:**

Because the proposed method uses SLINK to simulate the segregation process, it benefits from its flexibility: the trait may be qualitative with the possibility of defining different liability classes (which allows for the simulation of gene-environment interactions or even the simulation of multi-locus effects between unlinked susceptibility regions) or it may be quantitative and normally distributed. In particular, this implementation is the only one available that can generate a large number of marker loci conditional on the set of observed quantitative trait values in pedigrees.

## Background

In recent literature, algorithms and software were developed to simulate the segregation process of marker loci that are in linkage disequilibrium (LD), some allowing for the possibility for the markers to also be associated with a trait [1-3]. These tools were developed to answer important methodological questions and to help determine empirically the properties of family-based statistical tests, and they should be well received especially now that whole genome association scans have become an affordable reality [4,5]. There is an increasing body of literature on the benefits of using haplotypes for the mapping of susceptibility genes for complex traits (see [6] for a review), and guidelines on the choice of sample size and on the power of haplotype association studies are available for samples of unrelated individuals [7,8]. There is still room for significant advances in haplotype-based mapping methods; flexible and efficient simulation tools are necessary for evaluation of promising statistical methods or designs.

Most recently, the software SimPed [3] has been introduced to simulate the segregation and recombination processes of marker data in families of arbitrary structures. SimPed allows for the markers, or subsets of markers, to be in LD. The recombination process takes place under a genetic map that is constant between the two sexes. Segregation is performed under the rules of Mendelian inheritance, or, equivalently, under a null genetic model (no linkage and no association with any trait loci). SimPed is designed to evaluate the properties of statistical methods under the null genetic model.

The SIMLA software [1,2] has recently been extended to account for covariates and interactions in the etiology of the simulated disease or trait. Just like SimPed, the algorithm relies on "gene dropping" to segregate alleles or haplotypes (that may or may not include trait loci) starting from the founders down the lines of a pedigree structure with varying sibship sizes, but that includes at most four founders. Trait values are simulated according to the desired genetic model and pedigrees may be ascertained on conditions set by the user. SIMLA can be used to evaluate the power of statistical methods, under a wide variety of genetic models, for simple and constant pedigree structures.

An advantage of SIMLA over other gene dropping simulation tools (such as SimPed or SimM [9]) is that it conveniently combines the simulation of markers and trait values in a single tool (whereas, using the other two programs, segregation of a trait locus and flanking markers would have to be followed by the simulation of trait values with the help of an outside tool). This allows for the segregation of the trait locus or loci first, followed by the simulation of trait values. Then, only if the pedigree meets the conditions for ascertainment will the segregation process and the recombination process (under a sex-equal genetic map) of the complete marker data take place, conditional on the descent path of the chromosomes at the trait locus or loci. This strategy represents a gain in efficiency, especially for diseases with low prevalence, for which it is expected that only a small fraction of replicates can be ascertained.

In contrast to these gene dropping simulation tools, SLINK [10,11] (or, preferably, the faster C version of SLINK, FastSLINK [10-12]) generates marker data in families conditional on the observed trait values and conditional on already observed genotypes, if any, in an iterative fashion, using likelihood methods [13]. The markers and the trait locus may be either in linkage equilibrium (LE) or in LD with each other (the LD option has rarely been used in the literature, see [3,14-16] for applications). One of the unique features of FastSLINK is that it can generate data conditional on observed quantitative trait values, as long as the trait is normally distributed. One limitation of FastSLINK is that it can only accomodate a handful of markers (including the trait locus): the order of complexity of the algorithm described in Ott [10] scales exponentially with the number of markers. To help circumvent this restriction, I describe how FastSLINK can be used as part of a two-step algorithm to simulate the segregation process of a large number of marker loci linked to a trait locus and conditional on trait values, with the possibility for any subset of consecutive loci (that may or may not include the trait locus) to be in LD. The general ideas behind what follows were briefly described in [17] (the main reference for the SIMULATE software), but, to my knowledge, never implemented.

## Implementation

Given a pedigree, a set of observed trait values, and a genetic model that translates genotypes into trait values, the following implementation relies on FastSLINK to simulate the trait locus genotypes conditional on the observed trait values. Since FastSLINK can generate LD between a trait locus and a marker locus, it is possible to obtain in the output the simulated genotypes at the trait locus (which are normally only stored internally) simply by simulating a marker locus that is in perfect LD with the trait locus (with coefficient of determination *r*^2 ^= 1) and at the same genetic position. At the same time, a perfectly informative marker (all of the founders in the pedigree have different alleles and are heterozygous) is generated at the same genetic position as the trait locus; this marker serves to describe the segregation path of the chromosomes in the pedigree, and is known hereafter as the descent marker. The descent marker allele frequencies are irrelevant, and the marker is generated in LE with the trait locus. In the output of FastSLINK, the alleles of the genotypes are ordered in such a way that the phases of the chromosomes (or haplotypes) are fully known; thus, the descent marker provides complete information about the identity by descent state between any two trait locus alleles in the pedigree.

Then, in a second step, using a second program, genotypes for a large number of marker loci (a number of which may be in LD in an arbitratry number of "blocks" of LD) are simulated for all individuals in the pedigree. First, chromosomes in the founders are generated according to user-specified alleles and haplotype frequencies (when LD is desired) and conditional on the trait locus allele already found on the chromosome (to allow for the possibility of LD between markers and trait loci). Then segregation of the chromosomes at the position of the trait locus follows the path of the descent marker, while recombination occurs on both sides of the trait locus, according to a user-specified genetic map (sex-specific or sex-equal), without interference.

**Figure 1:**
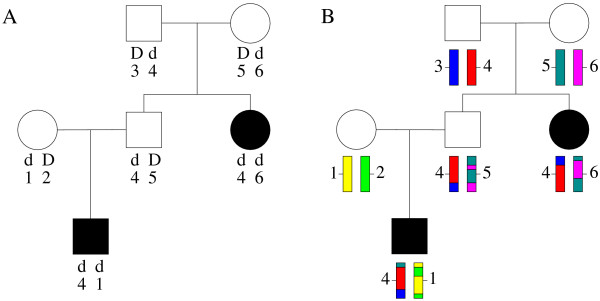
**A two step implementation**. (A) First step: simulating genotypes at the trait locus (with two alleles, *D *and *d*, the latter being the high risk allele) conditional on the observed trait values, and genotypes at a perfectly informative marker (the descent marker) at the same genetic position as the trait locus. Genotypes are phased. Individuals in black are affected; individuals in white are unaffected. (B) Second step: simulating haplotypes in the founders, allowing for the possibility for markers to be in LD with the trait locus (here warmer colors are associated with high risk alleles) followed by segregation of the chromosomes according to the descent marker and recombination on both sides of the trait locus (at the position of the descent marker) allowing for recombination to occur under sex-specific maps.

Figure 1 illustrates the two steps of the above algorithm.

Even though there is no serious limit to the size of the pedigrees that FastSLINK can handle, there is a limit on the number of alleles a marker can have: the implementation of FastSLINK uses a bitshift operator to shift a long integer by a number of bits that corresponds to an allele number. Thus, for most computers the total number of alleles a marker may have cannot exceed 32, which restricts the above implementation to pedigrees having at most 16 founders, which is still four times as much as the number of founders SIMLA handles.

The second step of the procedure described above has been implemented in a C++ program named SUP (Slink Utility Program; see Additional Files).

## Results and discussion

**Figure 2:**
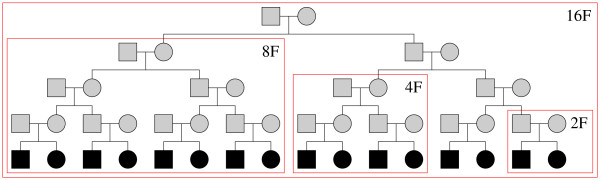
**One large pedigree and three sub-pedigrees used for simulations**. Pedigrees are named after the number of founders they contain. Individuals in black are affected; individuals in white are of unknown disease status.

The performance of the combination of FastSLINK and SUP to simulate genetic data linked to, or linked to and in LD with, a susceptibility locus was compared with that of SIMLA v3.1 and ALLEGRO v1.2c [18], which are to my knowledge the only two other software that can generate a set of replicates under a non-null genetic model for more than just a few markers (while SIMLINK [19] simulates marker data conditional on observed trait values, it is restricted by design to no more than two markers). Of these, only SIMLA can simulate LD. Table 1 summarizes the main characteristics of each software. Four pedigree structures were used for simulations, illustrated in Figure 2. The last generation consists of *F*/2 sib-pairs, where *F *is the number of founders in the pedigree. All individuals in the last generation were assumed to be affected; the affection status of all other individuals was assumned to be unknown, and thus free to vary.

**Table 1:** Characteristics of three software that simulate marker data under non-null genetic models.

	SLINK/SUP	SIMLA	ALLEGRO
Simulates on pedigrees as they have been collected	Yes	No	Yes
Simulates marker data conditional on			
1) observed affection status	Yes	Yes	Yes
2) observed affection status and observed exposure/liability class	Yes	No	No
3) observed quantitative trait values	Yes	No	No
			
Simulates LD between			
1) marker loci	Yes	Yes	No
2) marker and trait loci	Yes	Yes	No
			
Simulates values for			
1) affection status	Yes	Yes	No
2) environmental exposure	No	Yes	No
3) quantitative trait	Yes	Yes	No
4) covariates	No	Yes	No
			
Simulates multi-locus susceptibility between			
1) unlinked loci	Yes (indirectly)	Yes	No
2) linked loci	No/Future	Yes	No
			
Simulates under sex-specific maps	Yes	No	Yes
			
Simulates X-linked genetic data	Yes/Future	No	Yes
			
Simulates upon pedigrees with loops	Few	No	Yes
			
Pedigree restrictions	16 founders	4 founders	< 31 bits^†^

Some features are planned future extensions of SUP. ^†^The number of bits is defined as twice the number of non-founders minus the number of founders.

One susceptibility locus or two interacting ones were simulated for a dichotomous trait. In the case of one susceptibility locus, the high-risk allele frequency was taken to be 15%, and penetrances were chosen under a multiplicative model such that the relative risk for the heterozygote at the trait locus is 1.5 (2.25 for the high-risk homozygous). In the case of two susceptibility loci, a two-locus interaction threshold effect was simulated (see e.g. [20]): carriers of one or more high risk alleles at either or both trait loci increases the risk by 2.25 as opposed to non-carriers. High-risk alleles were taken to be 15% at each locus. Because the time it takes SIMLA to generate replicates of a disease status highly depends on its prevalence [2], the prevalence was evaluated at both 5% and 10%.

In all cases, two chromosomes each having 6 or 6000 markers were simulated. If only one susceptibility locus was simulated, then the second chromosome was generated under a null model. When simulating under LE, each marker had four equifrequent alleles. When simulating under LD, only 6 bi-allelic markers were simulated on each chromosome (the default limit of SIMLA) forming the 12 haplotypes found in Table 2 (identical for both chromosomes). When present, the high risk allele, or alleles in case of multi-locus susceptibility, were taken to lie exclusively on the second haplotype shown in Table 2.

**Table 2:** Haplotypes and their frequencies for simulations and validation. When simulating under a non-null genetic model, the high-risk allele lies exclusively on the second haplotype. For validation purposes, haplotype frequencies are compared between software in samples of 1000 affected sib-pair families, averaged over 25 runs, under a non-null genetic model.

Marker haplotype	Population frequency	Estimated frequency of haplotype in LD with trait locus	
		SLINK/SUP	SIMLA
1-1-1-1-1-1	0.5	0.463	0.461
1-1-1-1-1-2	0.25	0.305	0.308
1-1-1-1-2-2	0.125	0.115	0.116
1-1-1-2-2-2	0.0625	0.0592	0.0578
1-1-2-2-2-2	0.0312	0.0293	0.0292
1-2-2-2-2-2	0.0156	0.0143	0.0143
2-1-1-1-1-1	0.0078	0.00764	0.00716
2-2-1-1-1-1	0.0040	0.00352	0.00376
2-2-2-1-1-1	0.0020	0.00188	0.00208
2-2-2-2-1-1	0.0010	0.00048	0.00060
2-2-2-2-2-1	0.0005	0.00016	0.00008
2-2-2-2-2-2	0.0004	0.00001	0.00004

While FastSLINK can only generate a single trait locus in a single run, it can be used to simulate multi-locus susceptibility between unlinked loci in multiple runs: in the case of two loci, given a set of two-locus penetrances, the marginal penetrances can be calculated for one of the loci and, given the possible genotypes at that locus, conditional penetrances can be calculated for the second trait locus (see [21] for details). Then, genotypes at the first locus are simulated in a first run of FastSLINK using the marginal penetrances. These genotypes are then used to define different liability classes, each corresponding to a different set of penetrances (the conditional ones). The second trait locus is then simulated in a second run conditional on the observed traits and on the genotype-defined liability classes. This procedure cannot be acheived with ALLEGRO, as the trait locus genotypes are not available in the output and LD cannot be simulated between the trait locus and a marker locus.

Simulation times are found in Table 3. These are estimated from the average over 25 runs on a Xeon 3.0 Ghz processor running Gentoo Linux. For FastSLINK/SUP and ALLEGRO, no significant time differences were found between the two prevalence values; for these two software, only the times corresponding to the 10% prevalence disease model are shown. It can be seen that ALLEGRO is superior when generating data linked to and in linkage equilibrium with a single trait locus (the only cases it can handle), but is not able to generate data for the largest pedigree. SIMLA is comparable in speed to FastSLINK/SUP when only two affected sibs are present, unless a large number of markers are to be generated. Otherwise FastSLINK/SUP is superior in time. When more individuals are affected (as in 4F), the dependence of SIMLA on the prevalence of the disease is marked. No significant time differences can be seen if the six markers are in LD with each other.

**Table 3:** Time in seconds to generate a sample of families of identical structure. The pedigree structures are named after the number of founders (see Figure 2). Markers and trait locus are taken to be in linkage equilibrium (LE) or disequilibrium (LD). A dash indicates that the software is unable to simulate upon the pedigree structure or the model. *K *is the prevalence of the disease.

Number of markers	Pedigree structure (sample size)	FastSLINK/SUP	SIMLA		ALLEGRO
			*K *= 0.10	*K *= 0.05	
2 × 6 LE	2F (1000)	1.4	1.1	1.4	0.4
	4F (1000)	10.9	25.4	196.7	1.1
	8F (100)	12.3	-	-	0.8
	16F (10)	27.3	-	-	-
					
2 × 6000 LE	2F (1000)	64.5	160.8	189.8	51.5
	4F (1000)	163.2	2494.0	18403.1	125.5
	8F (100)	44.8	-	-	28.4
	16F (10)	33.6	-	-	-
					
2 × 6 LD	2F (1000)	1.3	1.1	1.3	-
	4F (1000)	11.0	26.1	197.7	-
	8F (100)	11.8	-	-	-
	16F (10)	27.1	-	-	-
					
2 × 6 LD multi-locus susceptibility	2F (1000)	7.5	1.1	1.4	-
	4F (1000)	50.0	25.3	197.1	-
	8F (100)	25.4	-	-	-
	16F (10)	53.5	-	-	-

The multi-locus susceptibility scenario takes more than twice the time for FastSLINK/SUP to generate the replicates compared with the single trait locus cases, mostly because FastSLINK is much faster to create, say, 1000 replicates of a single family (in run 1, to simulate the first trait locus) than to create a single replicate of a set of 1000 families (in run 2, to simulate the second trait locus). SIMLA always generates genotypes at two trait loci, even if only one is desired (in which case the user must select a null genetic model for the second trait locus). It is thus not surprising to see similar times for the one or two trait locus scenarios. If the two trait loci lay on the same chromosome, then SIMLA's times would be slightly longer [2]. It is planned to extend SUP to allow for two trait loci to be linked.

To get an idea of the increase in time it takes FastSLINK in its standalone version to simulate an increasing number of marker loci, note that it took 8 seconds for FastSLINK to generate a *single *replicate of only 3 markers (4 alleles each, in LE, in addition to the trait locus) in family 2F, while this time increased to more than 85 minutes for 4 markers. It was not possible to simulate more loci with FastSLINK due to memory constraints.

SUP's execution time scales linearly with the number of markers that are in LE, and linearly with the number of haplotypes with non-zero frequency. For illustration purposes, it was able to handle 100000 markers in LE and all 2097152 possible haplotypes of 21 SNPs in LD (including a trait locus).

To show the validity of the replicates generated by FastSLINK/SUP, haplotype frequencies were estimated from 1000 replicates of family 2F, generated under the scenario of a single susceptibility locus, in LD with the markers, forming the haplotypes found in Table 2. As above, the high-risk allele is found in a frequency of 15%, the relative risk per high-risk allele is 2.25 and the prevalence is set to 10%. Haplotype frequencies were calculated using Haploview v3.2 [22], and averaged over 25 runs. From Table 2, it can be seen that haplotypes generated by FastSLINK/SUP in the parental generation are found in similar frequencies as those generated by SIMLA. The same can be said about the haplotype frequencies calculated from the replicates unlinked to the susceptibility locus, which are then similar to the population frequencies in Table 2 (data not shown). This shows that, conditional on trait values, SUP generates correct frequencies. Moreover, SUP generates details about the location and the meioses in which recombination events take place during the simulation. These details were used to confirm that the recombination process follows the genetic map (sex-specific or sex-equal) provided in the input (data not shown).

## Conclusion

In summary, FastSLINK is one of the most flexible simulation tool that exists: the complexity of the algorithm described in Ott [10] is linear with the number of individuals, thus the pedigree may be arbitrarily large, it may contain consanguinity loops (but with considerable increase in computing time [23]; SUP supports pedigrees with inbreeding loops, but requires Mega2's [24,25] ability to break and reconnect them), and the trait may be qualitative with the possibility of defining different liability classes (to account, for example, for age-dependent penetrances, or to allow for gene-environment interactions or even, as described above, gene-gene interactions), or it may be quantitative and normally distributed. Sex-linked traits and genotypes can be simulated with FastSLINK, but this is not yet supported by SUP. FastSLINK's most serious handicap is that it can only generate data for only few markers, with considerable increase in computing time as the number of markers increases. Combining FastSLINK with SUP in the way presented here resolves this major restriction. One unique feature of FastSLINK/SUP that cannot, to my knowledge, be achieved with any other software is the possibility of efficiently generating a large number of markers or haplotypes conditional on observed quantitative trait values and in LD with the trait locus. This allows, for example, for efficient evaluation and comparision of the power of haplotype-based tests of association with quantitative traits, in actual samples of pedigrees, or even unrelated individuals (by adding dummy parents to create artificial pedigrees, since FastSLINK only allows simulation in families), as they have been collected and phenotyped.

With SIMLA and SLINK or SLINK-based approaches available, investigators have access to a wide variety of options, including the possibility of simulating linkage disequilibrium with one or more trait loci, to conduct efficient and realistic family-based simulation studies.

## Availability and requirements

**Project name: **SUP: Slink Utility Program

**Project home page: **

**Operating systems: **Unix/Linux

**Programming language: **C/C++

**Other requirements: **FastSLINK , gcc 2.95.3 or higher, perl, csh/tcsh

**License: **GNU General Public Licence

## Supplementary Material

Additional file 1 Included with this manuscript are the source code for SUP and documentation on both steps of the implementation described here. It is intended to be compiled on a UNIX/Linux system. On a Windows environment, the simplest way is to install CYGWIN , a linux emulator. It compiles using gcc version 2.95.3 and higher. To install, type at the prompt % gunzip sup.tgz % tar -xvf sup.tar Go to the src/directory and compile by typing % make or % g++ *.cc -o sup The documentation can be found in the doc/directory. In there, scripts are included to help in the creation of input files. These scripts require perl and csh/tcsh. Click here for file
